# Adenylyl Cyclase α and cAMP Signaling Mediate *Plasmodium* Sporozoite Apical Regulated Exocytosis and Hepatocyte Infection

**DOI:** 10.1371/journal.ppat.1000008

**Published:** 2008-02-29

**Authors:** Takeshi Ono, Laura Cabrita-Santos, Ricardo Leitao, Esther Bettiol, Lisa A. Purcell, Olga Diaz-Pulido, Lucy B. Andrews, Takushi Tadakuma, Purnima Bhanot, Maria M. Mota, Ana Rodriguez

**Affiliations:** 1 Department of Medical Parasitology, New York University School of Medicine, New York, New York, United States of America; 2 Sanaria Inc., Rockville, Maryland, United States of America; 3 Department of Parasitology and Immunology, National Defense Medical College, Tokorozawa, Saitama, Japan; 4 Department of Microbiology and Molecular Genetics, New Jersey Medical School, Newark, New Jersey, United States of America; 5 Faculdade de Medicina da Universidade de Lisboa, Instituto de Medicina Molecular, Lisboa, Portugal; Albert Einstein College of Medicine, United States of America

## Abstract

Malaria starts with the infection of the liver of the host by *Plasmodium* sporozoites, the parasite form transmitted by infected mosquitoes. Sporozoites migrate through several hepatocytes by breaching their plasma membranes before finally infecting one with the formation of an internalization vacuole. Migration through host cells induces apical regulated exocytosis in sporozoites. Here we show that apical regulated exocytosis is induced by increases in cAMP in sporozoites of rodent (*P. yoelii* and *P. berghei*) and human (*P. falciparum*) *Plasmodium* species. We have generated *P. berghei* parasites deficient in adenylyl cyclase α (*AC*α), a gene containing regions with high homology to adenylyl cyclases. *PbAC*α-deficient sporozoites do not exocytose in response to migration through host cells and present more than 50% impaired hepatocyte infectivity *in vivo*. These effects are specific to *AC*α, as re-introduction of *AC*α in deficient parasites resulted in complete recovery of exocytosis and infection. Our findings indicate that *AC*α and increases in cAMP levels are required for sporozoite apical regulated exocytosis, which is involved in sporozoite infection of hepatocytes.

## Author Summary

Malaria is transmitted through the bite of an infected mosquito that deposits *Plasmodium* sporozoites under the skin. These sporozoites migrate from the skin into the circulation and then enter the liver to start a new infection inside hepatocytes. Sporozoites have the capacity to traverse mammalian cells. They breach their membranes and migrate through their cytosol. This process is required for infection of the liver and triggers the exposure of adhesive proteins in the apical end of sporozoites, a process that facilitates invasion of hepatocytes. We found that elevations of cAMP inside sporozoites mediate the exposure of adhesive proteins and therefore the infection process. Mutant sporozoites that do not express adenylyl cyclase, the enzyme that synthesizes cAMP, are not able to expose the adhesive proteins and their infectivity is reduced by half. Reinsertion of adenylyl cyclase gene in the mutant sporozoites recovers their capacity to expose adhesive proteins and to infect hepatocytes, confirming the specific role of this protein in infection. These results demonstrate the importance of cAMP and the exposure of adhesive proteins in sporozoites, but also show that *Plasmodium* sporozoites have other mechanisms to invade host hepatocytes that are not inhibited in the mutant parasites.

## Introduction

*Plasmodium*, the causative agent of malaria, is transmitted by the bite of infected mosquitoes that inoculate the sporozoite form of the parasite in the host. Sporozoites rapidly migrate to the liver, where they infect hepatocytes, replicate and develop into merozoites, the blood-stage form of the parasite. *Plasmodium* belongs to the phylum apicomplexa, a group of parasites that share conserved mechanisms of motility and cell invasion machinery [1]. Apical exocytosis is another common feature that has been characterized in *Toxoplasma* tachyzoites [2] and sporozoites from *Eimeria* [3], *Cryptosporidium* [4] and *Plasmodium* [5]. This process has been most extensively studied in *Toxoplasma* tachyzoites, where active invasion of host cells involves the secretion of transmembrane adhesive proteins from the micronemes, which congregate on the anterior surface of the parasite and bind host receptors to mediate apical attachment [6]. One of these adhesive proteins, MIC2, which plays a central role in motility and invasion [7] is closely related to *Plasmodium* Thombospondin-Related Anonymous Protein, TRAP (also known as Sporozoite Surface Protein 2, SSP2) [8], which is also exposed in the apical end of the parasite upon microneme exocytosis [5],[9] and is also required for *Plasmodium* sporozoite motility and invasion [10].

While in *Toxoplasma* tachyzoites microneme secretion is strongly up-regulated upon contact with the host cell, in *Plasmodium* sporozoites contact with host cells is not sufficient to activate this process and migration through cells is required to induce apical regulated exocytosis [9]. Sporozoites of different human and rodent *Plasmodium* species have the ability to migrate through host cells. Sporozoites enter and exit cells by breaching the plasma membrane of the traversed cell. This process results in sporozoites traversing host cells by moving through their cytosol without any surrounding membranes. Ultimately, sporozoites establish infection in a final hepatocyte through formation of a vacuole within which the parasite replicates and develops [9]. Migration through host cells induces apical exocytosis in *Plasmodium* sporozoites, resulting in the exposure of high concentrations of TRAP/SSP2 in the apical end of the parasite [9]. This process, similarly to *Toxoplasma* secretion of MIC2 [7], is thought to facilitate invasion of the host cell [9].

During migration through host cells sporozoites are not surrounded by any host membranes, and as a result, they are in direct contact with the cytosol of the host cell [11]. Incubation of *Plasmodium* sporozoites with a lysate of host cells activates apical exocytosis in the parasite, suggesting that host cell molecules induce the activation of exocytosis in migrating parasites [9]. We have studied the role of uracil nucleotides in sporozoite exocytosis, since these molecules induce exocytosis in other cellular systems [12] and are found in the cytosol of mammalian cells in high concentrations. We found that uracil and its derived nucleoside and nucleotides (UMP, UDP and UTP) at the physiological concentrations found in the cytosol of mammalian cells, activate apical regulated exocytosis and increase the infectivity of sporozoites [13]. Since sporozoites are in contact with the cytosol of the traversed host cells, it is likely that the high concentrations of uracil derivatives that they would encounter, probably participate in the activation of sporozoites during migration through cells. Addition of uracil derivatives *in vitro* induces apical regulated exocytosis within the first ten minutes after addition of the stimulus [13]. In certain mammalian cell types, UTP and UDP can activate signaling cascades by binding to P2Y receptors, which in turn can activate adenylyl cyclase and increase cyclic adenosine monophosphate (cAMP) levels. Activation of P2Y receptors by nucleotides leads to exocytosis in different cells from insulin release from pancreatic islet β cells to the release of histamine from mast cells [14].

Here we have analyzed the role of the cAMP signaling pathway in sporozoite apical exocytosis and infection. We found biochemical evidences indicating that increases in cAMP levels in sporozoites mediate apical regulated exocytosis, which activates sporozoites for host cell invasion. By creating a parasite line deficient in adenylyl cyclase α (ACα), we confirmed that the cAMP signaling pathway is essential to induce apical exocytosis, which is activated during migration through cells. In addition, this recombinant parasite provides a tool to determine the precise contribution of apical exocytosis to sporozoite infection. A role for migration through cells and apical regulated exocytosis in infection was proposed before [9], but it had been questioned in view of transgenic sporozoites that were able to infect cells *in vitro* without performing the previous migration step [15]. Here we show that apical regulated exocytosis contributes significantly to host cell invasion, but the parasite seems to have alternative mechanisms to establish successful infections in host cells.

## Results

To investigate the signaling pathways mediating *Plasmodium* sporozoite exocytosis, we used a mix of uracil and its derivatives (uridine, UMP, UDP and UTP) at the concentrations normally found in the cytosol of mammalian cells (described in Experimental Procedures), which induce exocytosis in sporozoites [13]. Apical regulated exocytosis has been characterized in *Plasmodium* sporozoites by the exposure of high concentrations of TRAP/SSP2 in the apical end of the parasite and also by the release of this protein into the medium [9]. We confirmed that exocytosis occurs at the apical end of the sporozoite by staining the trails left behind after gliding motility. Trails are always next to the posterior end because sporozoites move with their apical end in the front (Fig. S1).

**Figure 1:**
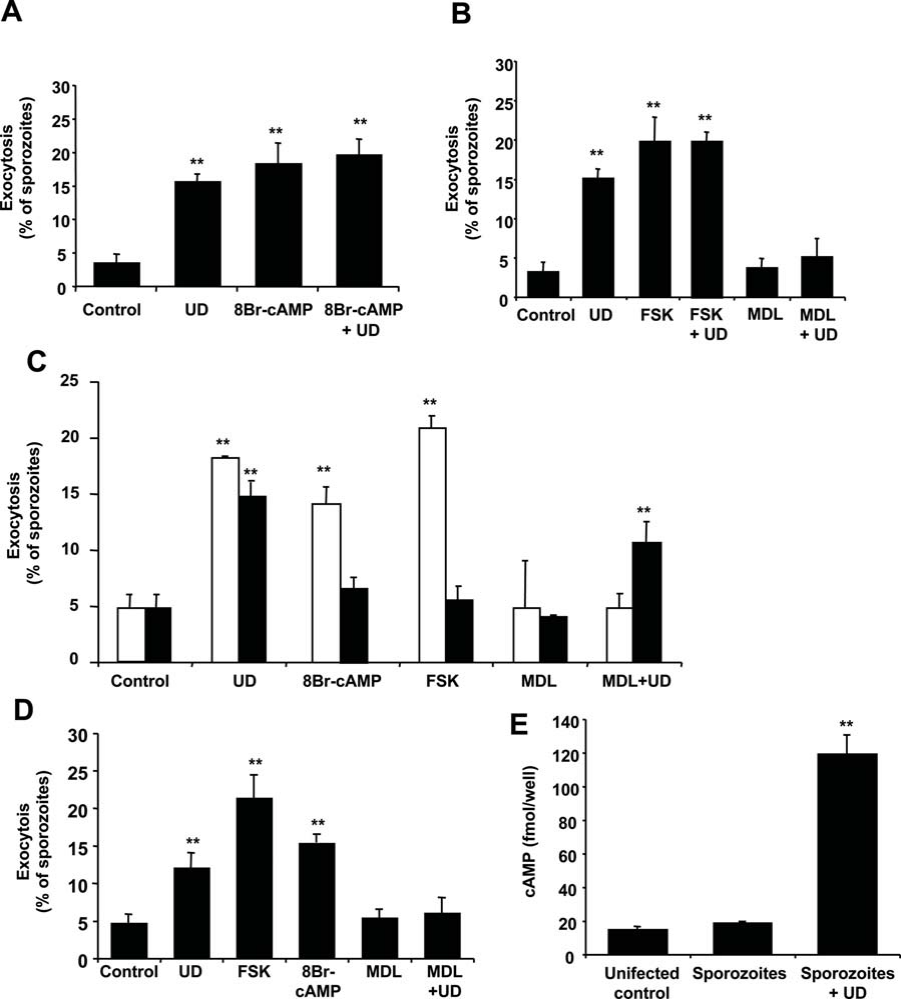
Increases in cytosolic cAMP induce *Plasmodium* sporozoite exocytosis. (A–B) *P. yoelii* sporozoites were pre-incubated for 15 min with 8Br-cAMP, forskolin (FSK) or MDL-12.330A to activate or inhibit adenylate cyclase respectively, followed by addition or not of uracil derivatives (UD). Sporozoites were incubated for 1 h before fixation and quantification of exocytosis. (C) *P. berghei wt* (white bars) or *spect 1*-deficient (black bars) sporozoites were pre-incubated with the different activators and inhibitors as in (A,B). (D) *P. falciparum* sporozoites were pre-incubated with the different activators and inhibitors as in (A,B). (E) Intracellular levels of cAMP in *P. yoelii* sporozoites incubated or not with uracil derivatives for 45 min. Same number of uninfected salivary glands were processed in a similar way and used as a control (uninfected). Results are expressed as mean of triplicates±SD. *, *p*<0.05; ** *p*<0.01 when compared to control by ANOVA.

We first investigated whether cAMP induces or modulates sporozoite regulated exocytosis by preincubating *P. yoelii* sporozoites with a membrane permeant analogue of cAMP. Exocytosis is quantified as the percentage of sporozoites that present a defined accumulation of extracellular TRAP/SSP2 in their apical end [9]. We found that 8Br-cAMP induces sporozoite exocytosis to a similar level than uracil derivatives. Addition of both stimuli to sporozoites did not increase the level of exocytosis (Fig. 1A), suggesting that both stimuli may be using the same pathway to induce exocytosis. As an alternative way to increase cytosolic cAMP in sporozoites, we used forskolin, an activator of the enzyme that synthesizes cAMP, adenylyl cyclase (AC). This treatment also induced apical regulated exocytosis in sporozoites (Fig. 1B). Incubation of sporozoites with MDL-12,330A, an inhibitor of AC [16] prevented activation of exocytosis by uracil derivatives (Fig. 1B). We confirmed that these treatments did not increased sporozoite lysis compared to control (Table S1 and Fig. S2).

Genetically manipulated sporozoites that are deficient in their capacity to migrate through cells (*spect*-deficient) infect hepatic cell lines *in vitro*, questioning the role of migration through cells in the activation of sporozoites for infection [15]. To analyze the exocytosis response of these sporozoites, we stimulated them with uracil derivatives or treatments that modulate cAMP levels. Incubation of *P. berghei wt* or *spect*-deficient sporozoites with uracil derivates induced apical regulated exocytosis. However, forskolin and 8-Br-cAMP did not induce exocytosis in *spect*-deficient sporozoites and MDL-12,330A only has a partial effect in the inhibition of exocytosis (Fig. 1C). These results suggest that, in contrast to *wt P. berghei* sporozoites, *spect*-deficient sporozoites do not use cAMP-mediated signaling pathways to activate exocytosis.

We have used the rodent malaria parasites *P. yoelii* and *P. berghei* as a model for *P. falciparum*, the human parasite responsible for the mortality associated with this disease. *P. falciparum* sporozoites also migrate through host cells [11], a process that induces apical regulated exocytosis in this species of the parasite [13]. Similar to the rodent parasites, uracil and its derivatives induce exocytosis in *P. falciparum* sporozoites [13]. We found that elevated cAMP levels also induce exocytosis in *P. falciparum* sporozoites and that exocytosis induced by uracil derivatives is inhibited by MDL-12,330A (Fig. 1D), suggesting that this pathway is conserved in the human and murine parasites.

To directly demonstrate that cAMP levels are increased in *P. yoelii* sporozoites in response to exocytosis-inducing stimuli, we measured cAMP concentration in sporozoites after incubation with uracil derivatives. Salivary glands dissected from uninfected mosquitoes and processed in a similar way, were used as negative control. We found that uracil derivatives significantly increase the levels of cAMP in sporozoites (Fig. 1E). No increases were found when control material from uninfected mosquitoes was stimulated with uracil derivatives (not shown).

**Figure 2:**
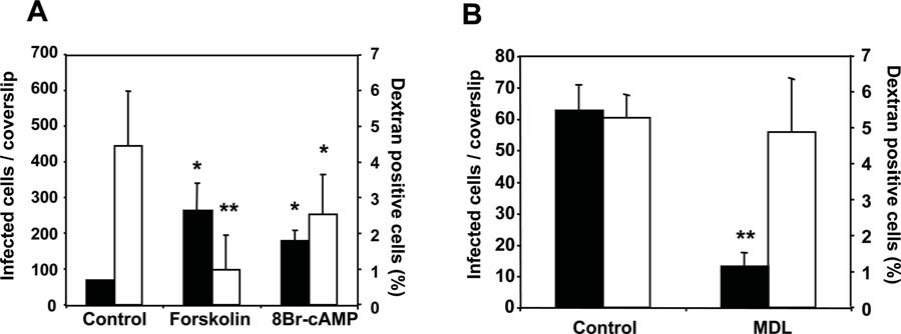
Stimulation of exocytosis increases sporozoite infection and decreases migration through host cells. *P. yoelii* sporozoites were pretreated with forskolin or 8Br-cAMP (A) or MDL-12.330A (B) before addition to monolayers of Hepa1-6 cells. Percentage of dextran-positive cells (white bars) and number of infected cells/coverslip (black bars) are shown as mean of triplicates±SD. *, *p*<0.05; ** *p*<0.01 when compared to control by ANOVA.

Migration through host cells induces sporozoite apical regulated exocytosis, which activates sporozoites for infection. Stimulation of exocytosis by other means, such as host cells lysate [9] or uracil derivatives [13], overcomes the need for extensive migration through cells and increases infection. To test whether stimulation of exocytosis by increases in intracellular cAMP in the sporozoite would also overcome the need for migration through host cells before infection, we incubated *P. yoelii* sporozoites with forskolin or 8Br-cAMP to induce regulated exocytosis before addition of sporozoites to intact Hepa1-6 cells. Migration through host cells is determined as the percentage of cells that are wounded by sporozoite migration and as a result become positive for a soluble impermeant tracer (dextran) [17]. We found an increase in the number of infected cells, indicating that stimulation of regulated exocytosis by cAMP in sporozoites increases their infectivity (Fig. 2A, black bars). In addition, activation of sporozoite exocytosis with increased cAMP levels reduces sporozoite migration through host cells, confirming that such extensive migration is no longer necessary when exocytosis is induced by elevations in the level of cAMP (Fig. 2A, white bars). These results indicate that cAMP-induced exocytosis contributes to the activation of sporozoites for infection.

Since sporozoites appear to activate the cAMP signaling cascade to stimulate apical regulated exocytosis, inhibition of cAMP production in sporozoites by MDL-12,330A, the inhibitor of AC, should decrease their infectivity. We actually found a significant reduction in their infectivity after treatment with this inhibitor (Fig. 2B). MDL-12.330A does not appear to have a toxic effect on sporozoites, since migration through cells was not affected (Fig. 2B).

We also observed that gliding motility of sporozoites is greatly decreased 18 to 24 min after addition of the exocytosis inducing stimulus (UD or forskolin), but not during earlier time points, while exocytosis is presumably occurring (0 to 8 min after addition of the stimulus) (Fig. S3).

**Figure 3:**
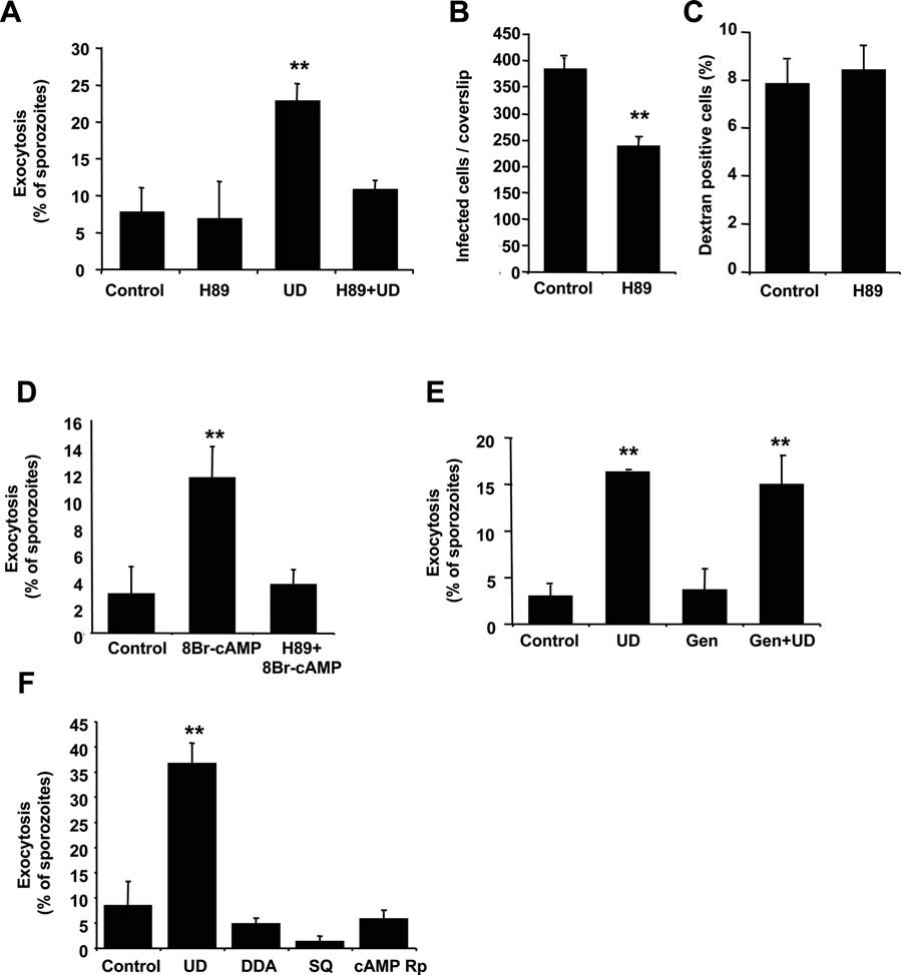
Treatment with an inhibitor of PKA reduces sporozoite exocytosis and infection. *P. yoelii* sporozoites were pre-incubated with H89 followed by addition of uracil derivatives to induce exocytosis (A) or followed by incubation with monolayers of Hepa1-6 cells to quantify infection (B) and migration though cells (C). (D) Sporozoites were pre-incubated with H89 before addition of 8Br-cAMP to induce exocytosis. (E) Sporozoites were pre-incubated with genistein (Gen) before addition of uracil derivatives. (F) *P. yoelii* sporozoites were pre-incubated with 2′, 5′-Dideoxyadenosine (DDA) or SQ22536 (SQ) to inhibit adenylyl cyclase activity or with cAMP Rp-isomer to inhibit PKA, before addition of uracil derivatives to induce exocytosis. Results are expressed as mean of triplicates±SD. * *p*<0.05; ** *p*<0.01 when compared to control by ANOVA.

The major downstream effector of cAMP is protein kinase A (PKA), a serine/threonine kinase that activates other kinases and transcription factors in the cell. This protein is likely to be present in *Plasmodium* because PKA activity has been detected in *P. falciparum* during the blood stage of the parasite [18],[19] and there is a gene sequence with high homology to PKA expressed in *P. falciparum* and conserved in all species of *Plasmodium* analyzed [20],[21], however no functional assays have yet determined the PKA activity of this putative protein. To investigate whether sporozoite exocytosis is mediated by PKA activity, we treated sporozoites with H89, a PKA inhibitor already shown to inhibit this kinase in a different stage of the parasite [18],[19]. We found that H89 inhibits sporozoite exocytosis induced by uracil derivatives (Fig. 3A), suggesting that this process is mediated by the activation of PKA. The infectivity of sporozoites pretreated with H89 is reduced, probably as a consequence of the inhibition of exocytosis (Fig. 3B), while parasite migration through host cells is not affected, confirming that H89 treatment is not toxic for sporozoites (Fig. 3C).

Activation of PKA should occur after cAMP has been generated in the signaling cascade. To analyze this step of the pathway, we pretreated sporozoites with H89 before increasing cAMP levels with the addition of 8Br-cAMP. As expected, we found that exocytosis was completely inhibited (Fig. 3D), suggesting that PKA is activated down-stream of cAMP. Incubation of sporozoites with genistein, an inhibitor of tyrosine kinases, did not affect regulated exocytosis (Fig. 3E), indicating that tyrosine kinases are not involved in the signaling cascade. In fact, no sequences with homology to tyrosine kinases have been found in the *Plasmodium* genome [20].

To strengthen the evidence that the cAMP signaling pathway mediates the activation of exocytosis in sporozoites and reduce the probability of inhibitors affecting exocytosis due to non-characterized effects of the drugs, we used alternative inhibitors with unrelated chemical structures from the ones used before to inhibit adenylyl cyclase and PKA. We found similar inhibitory results using 2′, 5′-Dideoxyadenosine or SQ22536, which inhibit adenylyl cyclase. The addition of a competitive inhibitor of cAMP (cAMP Rp-isomer), which inhibits PKA, also results in inhibition of apical regulated exocytosis in sporozoites (Fig. 3F).

Since cAMP signaling appears to mediate the activation of apical exocytosis, we searched for ACs in the malaria genome. Two different genes with high homology to ACs (ACα and ACβ) have been identified in *Plasmodium*. In particular, ACα was shown to have AC activity in *P. falciparum* [22],[23]. Interestingly, ACα genes from *Plasmodium*, *Paramecium* and *Tetrahimena* are closely related and their sequence includes a domain with high homology to K^+^ channels [23]. In *Paramecium*, where the purified AC protein also has K^+^ channel activity, generation of cAMP is regulated by K^+^ conductance [24]. It is thought that ACα presents a transmembrane K^+^-channel domain and an intracellular AC domain, which are functionally linked [25].

Since cAMP in *Plasmodium* sporozoites induces apical exocytosis, we first tested whether extracellular K^+^ is required for this process. In fact, sporozoites must remain in a high K^+^ environment during migration through cells, because the cytosol of eukaryotic cells has high concentrations of this ion [26]. The existence of K^+^ channels has been predicted for *Plasmodium* parasites from electrophysiological [27] and genomic sequence data [20].

**Figure 4:**
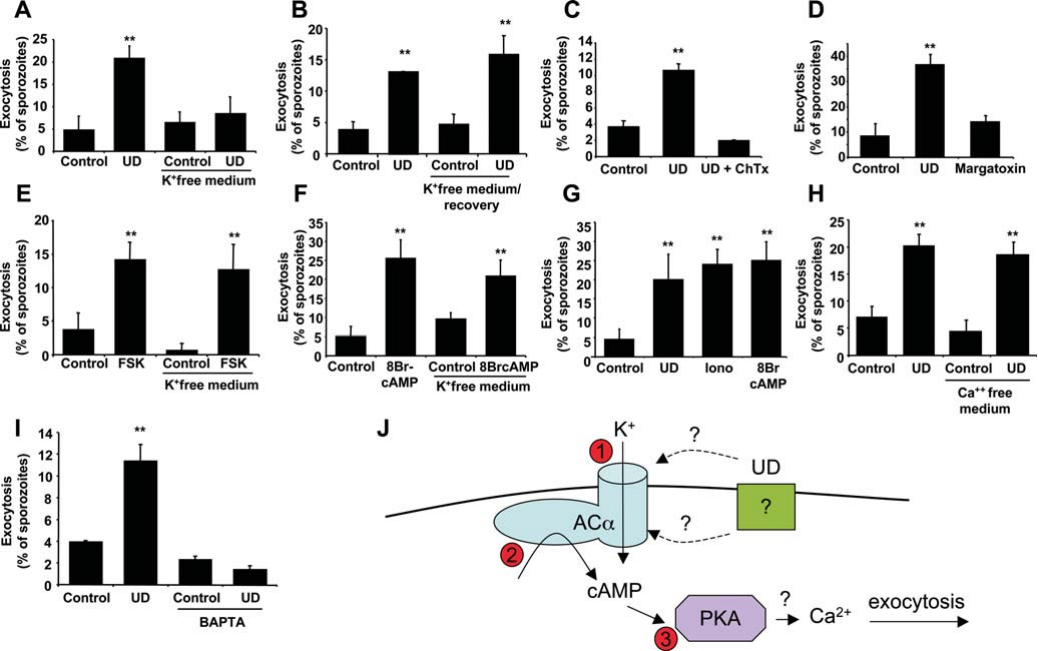
Extracellular K^+^ is required for sporozoite apical regulated exocytosis. (A) *P. yoelii* sporozoites were pre-incubated for 15 min in regular medium or K^+^-free medium before addition or not of uracil derivatives (UD) for 45 min. (B) Sporozoites were incubated with regular medium or K^+^-free medium for 45 min, followed by incubation in regular medium in the presence or absence of UD for another 45 min. (C,D) Sporozoites were pre-incubated with the K^+^-channel inhibitors charybdotoxin (C) or margatoxin (D) for 15 min before addition of UD for 45 min. (E,F) sporozoites were pre-incubated for 15 min in regular medium or K^+^-free medium before addition or not of forskolin (E) or 8Br-cAMP (F). (G) Sporozoites were incubated with UD, ionomycin or 8Br-cAMP for 45 min. (H) Sporozoites were pre-incubated for 15 min in regular medium or Ca^++^-free medium before addition or not of UD for 45 min. (I) Sporozoites were pre-incubated with the membrane permeant calcium chelator BAPTA-AM for 15 min before addition of UD for 45 min. Results are expressed as mean of triplicates±SD. ** *p*<0.01 when compared to control by ANOVA. (J) Possible model consistent with the results. UD activate directly or indirectly the K+ channel domain of ACα (1) and trigger the activation of AC activity (2). The increase in cAMP activates PKA (3), which leads to the activation of exocytosis.

To determine whether extracellular K^+^ is required for sporozoite exocytosis, we stimulated exocytosis in *P. yoelii* sporozoites in regular medium (containing K^+^) or in K^+^-free medium. We found that exocytosis stimulated with uracil derivatives was inhibited in K^+^-free medium (Fig. 4A). To confirm that sporozoites were not impaired by the incubation in K^+^-free medium, we transferred sporozoites to regular medium after the K^+^-free medium incubation. We found that exocytosis in these sporozoites was similar to exocytosis in sporozoites that were never incubated in K^+^-free medium (Fig. 4B).

Exocytosis was inhibited when sporozoites were pre-incubated with different K^+^-channel inhibitors (Fig. 4C,D), suggesting that K^+^ is required for the activation of exocytosis. We also analyzed the requirement for extracellular K^+^ in sporozoite exocytosis induced by 8Br-cAMP or forskolin. We found that in these cases extracellular K^+^ is not required (Fig. 4E,F), suggesting that extracellular K^+^ is required upstream cAMP in the signaling cascade. Removal of K^+^ from the medium may alter the electrochemical gradient of sporozoites affecting UD-induced exocytosis. However, since the response to forskolin and 8Br-cAMP in K^+^ free medium is not affected, it suggests that the sporozoite exocytosis pathway is perfectly functional in the absence of extracellular K^+^. Also, the viability (Table S1) and capacity of exocytosis response (Fig. 4B) of sporozoites after this treatment was found to be unaffected.

A Ca^++^ ionophore can induce apical regulated exocytosis in *P. yoelii* [9], suggesting that Ca^++^ signaling may be involved in exocytosis. We first compared the magnitude of the cAMP-induced to the Ca^++^-induced exocytosis, finding similar results (Fig. 4G). To study whether Ca^++^ is also involved in the signaling induced by UD, we induced exocytosis with UD in Ca^++^-free medium. We found that exocytosis is not inhibited in Ca^++^-free medium (Fig. 4H), suggesting that extracellular Ca^++^ is not required for this process. However, we found a strong inhibition of exocytosis when sporozoites were incubated with a membrane-permeant Ca^++^ chelator, suggesting that intracellular Ca^++^ is required for exocytosis (Fig. 4I). A possible model for the signaling mediating exocytosis is proposed (Fig. 4J).

Since *Plasmodium* sporozoite regulated exocytosis requires both extracellular K^+^ and cAMP, we decided to test whether *AC*α is involved in the process of sporozoite exocytosis and activation for infection by producing recombinant parasites deficient for this enzyme. We identified the sequence encoding *PbAC*α, the *P. berghei* orthologue of *PfAC*α, in the PlasmoDB database (http://www.plasmoDB.org/). Complete *PbAC*α sequences were retrieved from Sanger sequencing genomics project (http://www.sanger.ac.uk/). We found that *PbAC*α is 60% identical to *PfAC*α at the amino-acid level of the full-length predicted protein, and 79% in the AC catalytic domain.

**Figure 5:**
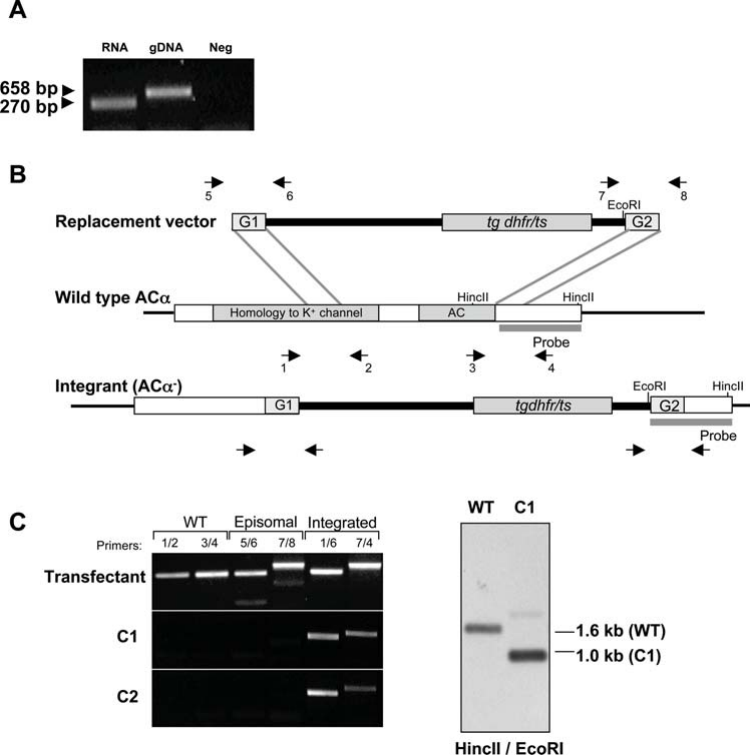
Generation of *PbAC*α- parasite lines. (A) RNA from WT *P. berghei* sporozoites was reverse transcribed into cDNA and used as template to amplify *AC*α. Water was used as negative control (Neg) and wild type *P. berghei* genomic DNA (gDNA) as positive control. (B) Schematic representation of the *AC*α locus and the replacement vector. Correct integration of the construct results in the disrupted *AC*α gene as shown. Arrows indicate the position of the primers used for PCR in C. (C) Disruption of *AC*α was shown by PCR (*left*) and by Southern analysis (*right*). PCR on DNA of WT transfected population (before cloning) and *PbAC*α- clones (C1 and C2) results in the amplification of two 0.7-kb WT fragments and a 0.8 and a 0.9-kb disrupted fragments when using the primers indicated in (B). Genomic Southern blot hybridization of WT and the *PbAC*α- C1. The probe used for hybridization is represented in B. Integration of the targeting plasmid causes reduction in size of a 1.6-kb fragment in WT parasites to a 1.0-kb fragment in the *PbAC*α- parasites. Similar results were found for *PbAC*α- C2.

Microarray analysis had detected expression of *PfAC*α in sporozoites [28]. To analyze the expression of *PbAC*α, we isolated mRNA from *P. berghei* sporozoites and performed reverse transcription followed by PCR. We also found expression of this gene in sporozoites (Fig. 5A). Thus, we decided to pursue a targeted gene disruption at the blood stages to study the importance of *AC*α for the *Plasmodium* pre-erythrocytic life cycle stages. We created two independent cloned lines of *P. berghei* parasites that are deficient in *AC*α (*PbAC*α-) by using targeted disruption of the *AC*α gene through double crossover homologous recombination (Fig. 5B). *PbAC*α-deficiency of the mutant parasites was confirmed by RT-PCR and Southern Blotting (Fig. 5C).

**Figure 6:**
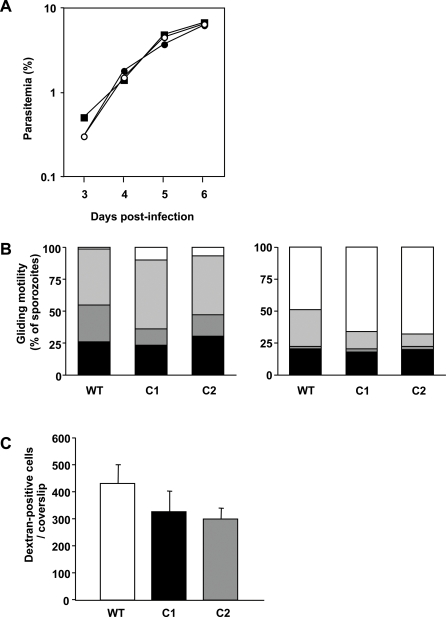
*PbAC*α- has normal blood-stage growth rates and sporozoite motility. (A) Growth curves of *P. berghei WT* (black squares), *PbAC*α- C1 (black circles) and C2 (white circles) in mice. (B) Gliding motility of sporozoites from *WT*, *PbAC*α- C1 and C2 in the presence (right panel) or absence (left panel) of mouse albumin. Percentage of sporozoites that do not glide or do less than a complete circle (black bars), gliding sporozoites exhibiting 1 (dark gray bars), 2 to 10 (light gray bars), or >10 (white bars) circles per trail. (C) Migration through Hepa1-6 cells was measured as the number of dextran positive cells per coverslip. The difference between C1 or C2 and *WT* is not significantly different (*p*>0.05).

We examined the phenotype of *PbAC*α- parasites during the *Plasmodium* life cycle. We compared the two *PbAC*α- lines with *WT P. berghei* parasites also cloned independently. *PbAC*α- parasites were indistinguishable from *WT* parasites in growth during red blood cell stages in mice (Fig. 6A). We next analyzed parasite growth in the mosquito by determining oocyst development and sporozoite salivary gland invasion. Similar oocyst and salivary gland sporozoite numbers were obtained for *PbAC*α- and the *WT* control, indicating that *PbAC*α is not involved in oocyst development and sporozoite salivary gland invasion (Table 1).

**Table 1:** 

	Midgut		Salivary glands
	Number of oocysts per infected mosquito (day 11)	Percentage of infected midguts (day 11)	Number of salivary gland sporozoites per mosquito (day 18)
WT	36	76	3,157
C1	37	80	3,653
C2	33	80	3,333

Gliding motility, the characteristic form of substrate-dependent locomotion of salivary gland sporozoites, was unaffected in *PbAC*α- parasites. Stimulation of gliding motility with albumin [29] was also similar in *WT* and *PbAC*α- sporozoites (Fig. 6B). We also tested whether deletion of the *AC*α gene affect sporozoites ability to migrate through cells. We found that the cell-traversal activity of *PbAC*α- sporozoites was slightly lower, but not significantly different from *WT* sporozoites (Fig. 6C).

**Figure 7:**
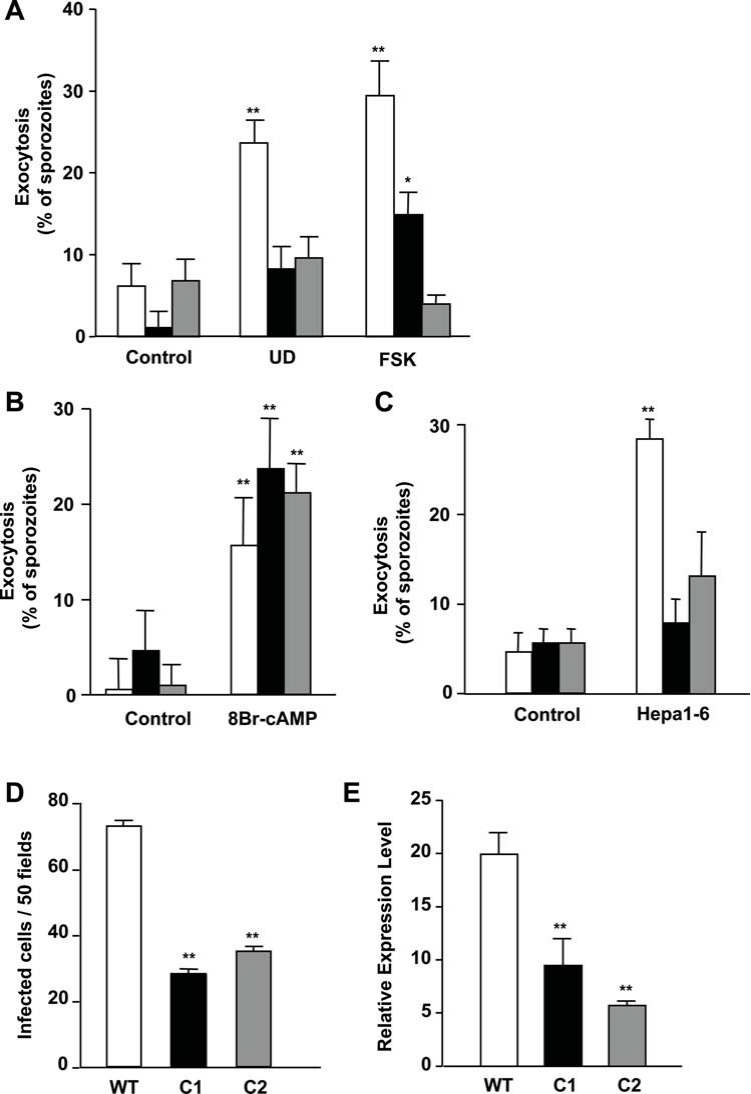
*PbAC*α- sporozoites have defective exocytosis and infection. Exocytosis and infectivity of *P. berghei WT* (white bars), *PbAC*α- C1 (black bars) and C2 (gray bars) sporozoites was analyzed. (A, B) Sporozoites were incubated or not with uracil derivatives (UD) or forskolin (FSK) (A) or 8Br-cAMP (B) for 1 h before fixation and quantification of exocytosis. (C) Sporozoites were added to filter insets containing confluent Hepa1-6 cells and collected on empty coverslips placed underneath the filters in the lower chamber. Percentage of sporozoites in coverslips showing apical-regulated exocytosis is shown. (D) Infection of Hepa1-6 cell by sporozoites *in vitro* was determined by counting the number of infected cells after 24 h incubation. (E) Infection of mice was determined by real-time PCR amplification of 18S rRNA in the liver 40 h after inoculation of sporozoites.

We then tested whether apical regulated exocytosis was affected in *PbAC*α-sporozoites. Activation of exocytosis by the mix of uracil derivatives or by forskolin, was greatly reduced in the two different clones of *PbAC*α- sporozoites analyzed (Fig. 7A). Addition of a membrane permeant analogue of cAMP (8-Br-cAMP), which induces exocytosis in *WT* parasites, also stimulated exocytosis in *PbAC*α- sporozoites (Fig. 7B). This result indicates that all sporozoite components required for exocytosis downstream of cAMP are functional in *PbAC*α- sporozoites; however, the lack of *AC*α inhibits proper response upon activation with uracil derivatives or activators of AC activity. Migration through host cells induces apical regulated exocytosis in *Plasmodium* sporozoites [9]. To confirm that *AC*α is also required for exocytosis stimulated by migration through hepatocytes, we measured the response of *WT* and *PbAC*α- sporozoites after migration through Hepa1-6 cells. We found that regulated exocytosis was not activated in sporozoites deficient in *AC*α (Fig. 7C).

To examine the role of apical regulated exocytosis and *AC*α in sporozoite infection, we first analyzed the infectivity of *PbAC*α- sporozoites *in vitro* using Hepa1-6 cells. We found that *PbAC*α- sporozoites are approximately 50% less infective than *WT* sporozoites (Fig. 7D). As the infectivity of *Plasmodium* sporozoites can be noticeably different depending on each particular mosquito infection, we repeated the experiment using sporozoites from three different batches of infected mosquitoes. Similar results were found, confirming that *PbAC*α- sporozoites have reduced infectivity in hepatocytes (not shown).

We also tested the infectivity of *PbAC*α- parasites *in vivo* in C57/Bl6 mice, which are highly susceptible to infection by *P. berghei* sporozoites [30]. To quantify the infectivity of *PbAC*α-, we used real time PCR to measure parasite load in the liver by determining the levels of the parasite-specific 18 S rRNA [31]. Remarkably, 50% decrease of parasite rRNA was detected by this method (Fig. 7E). We repeated the experiment using sporozoites from three different batches of infected mosquitoes finding similar results (not shown). These results suggest that *Plasmodium* sporozoites use apical regulated exocytosis to infect host cells and that *AC*α is an important protein involved in *Plasmodium* liver infection.

**Figure 8:**
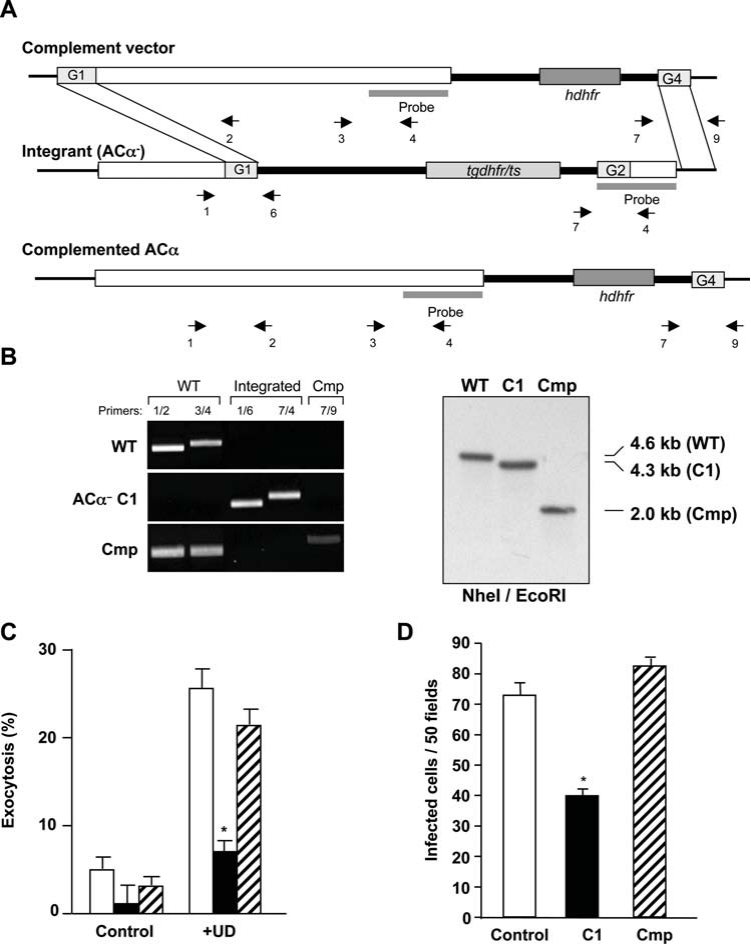
*PbAC*α- complemented sporozoites recover the *WT* phenotype. (A) Schematic representation of the complement replacement vector, the *AC*α- disrupted locus and the complemented *AC*α locus. Correct integration of the construct results in the reconstitution of the disrupted *AC*α gene as shown. Arrows indicate the position of the primers used for PCR in B. (B) Complementation of *AC*α was shown by PCR (*left*) and by Southern analysis (*right*). PCR on DNA of *WT*, *PbAC*α- C1 and complemented *AC*α (Cmp) results in the amplification of a fragment of 1 kb when using the primers indicated in (A). Genomic Southern blot hybridization of *WT*, *PbAC*α- C1 and complemented *AC*α. The probe used for hybridization is represented in A. Integration of the complementation plasmid causes reduction in size of a 4.3-kb fragment in *PbAC*α- C1 parasites to a 2.0-kb fragment in the *AC*α^−^ complemented parasites. (C) Exocytosis of WT (white bars), *PbAC*α- C1 (black bars) and complemented *AC*α (stripped bars) sporozoites in response to uracil derivatives (UD). (D) Infection of Hepa1-6 cells *in vitro* by WT, *AC*α- C1 (black bars) and complemented *AC*α (stripped bars) sporozoites was determined by counting infected cells 24 h after addition of sporozoites. * significant difference (*p*<0.01, ANOVA) compared to *WT* and complemented *AC*α.

To confirm that the phenotype observed in the *PbAC*α- sporozoites is caused specifically by depletion of the *PbAC*α gene, we complemented one of the *PbAC*α- parasite lines with *AC*α. The correct replacement event was confirmed by PCR and Southern blot hybridization (Fig. 8A). No differences were found between the complemented parasite line and *WT* or *PbAC*α- parasites during blood stage infection in mice or in mosquito oocyst development and salivary gland sporozoite numbers (not shown). We found that apical regulated exocytosis response to uracil derivatives was recovered in the complemented sporozoites (Fig. 8B). The infectivity of sporozoites was restored by complementation of the *PbAC*α gene (Fig. 8C), confirming the role of PbACα in sporozoite exocytosis and infection.

## Discussion

The role of exocytosis of apical organelles in invasion of host cells has been extensively studied in *Toxoplasma* tachyzoites. Our knowledge of *Plasmodium* sporozoite exocytosis and infection is less advanced, as this parasite stage can only be obtained by dissection of infected mosquitoes, and this procedure provides limited numbers of sporozoites. Sporozoite purification methods have been recently developed (S. L. Hoffman, personal communication) allowing us to use highly purified *P. falciparum* sporozoites in our studies. Gene deletion technology has opened the possibility of dissecting the role of complex pathways into their individual protein components. Using a rodent malaria model we have first identified that the cAMP signaling pathway is involved in *Plasmodium* sporozoite exocytosis. The similar response observed in *P. falciparum* sporozoites suggests that the cAMP-dependent signaling pathway leading to exocytosis is conserved in the human parasite. Based on these results, we have generated a transgenic parasite that is deficient in an essential protein in the cAMP signaling pathway. This approach allowed us to evaluate the role of apical regulated exocytosis in hepatocyte infection by sporozoites *in vitro* and *in vivo* using a mouse model.

Regulated exocytosis in mammalian cells is frequently triggered by an elevation of intracellular Ca^2+^ levels and is modulated by cAMP, which acts synergistically with Ca^2+^, but cannot induce exocytosis by itself. However, in some specific cell types exocytosis is triggered solely by elevations in cAMP concentrations [32]. Increases in cytosolic Ca^2+^ induced with ionophores can induce exocytosis in *Plasmodium* sporozoites [9], suggesting that Ca^2+^ stimulation is also sufficient to induce this process. The signaling pathways of Ca^2+^ and cAMP are interrelated inside eukaryotic cells [33]. In particular, in *P. falciparum* blood-stages, a cross-talk between Ca^2+^ and cAMP has been observed, where increases in cAMP induce the elevation of intracellular Ca^2+^ concentrations through the activation of PKA [18]. Our results suggest that the cAMP and Ca^2+^ pathways are also interconnected in the sporozoite stage and that intracellular, but not extracellular Ca^2+^, is required for exocytosis.

When exocytosis is inhibited by the AC or the PKA inhibitors, the reduction in sporozoite infectivity is comparatively lower than the reduction in exocytosis. Similar results were obtained with the *PbAC*α- sporozoites, where exocytosis is reduced to background levels, but infection is reduced by 50%. Taken together these results suggest that sporozoites have alternative pathways to invade host hepatocytes that do not require apical regulated exocytosis. However, we cannot exclude the possibility that low levels of exocytosis that cannot be detected in our assays still occur in the *PbAC*α- sporozoites and are sufficient to mediate infection of hepatocytes.

The analysis of host cell molecules required for sporozoite infection has provided evidence that sporozoites use more than one unique pathway to achieve hepatocyte infection [34], suggesting that sporozoites may take advantage of this phenomenon to overcome polymorphisms in host receptors or to escape from immune mechanisms inhibiting one particular pathway of infection.

We had previously observed that activation of sporozoite exocytosis increases their infectivity and reduces the need for migration through cells [9]. Here we confirmed that activation of exocytosis by cAMP-mediated pathways increases exocytosis infectivity reducing migration through cells. Accordingly, inhibitors of this pathway inhibit sporozoite exocytosis and decrease their infectivity. Interestingly, *spect*-deficient sporozoites, which do not migrate through host cells [15], responded to uracil derivatives but were not able to respond to either an activator of AC or to a permeant analogue of cAMP, suggesting that cAMP-induced signaling leading to exocytosis is different in these mutant sporozoites. The positive exocytosis response observed in the presence of the inhibitor of AC, suggests that these parasites are able to respond to uracil derivatives by activating cAMP-independent pathways that are not normally activated in *wt* sporozoites, where cAMP is required for exocytosis. It is still not clear how this relates to their impaired capacity to migrate through cells, but suggests that they may up-regulate the alternative mechanisms that are independent of migration through cells and exocytosis to infect hepatocytes. These results are consistent with the concept that sporozoites can use alternative pathways to invade hepatocytes, as the infection experiments with *PbAC*α- sporozoites suggest.

Apical regulated exocytosis in the transgenic parasites deficient in *AC*α is dramatically decreased in response to uracil derivatives or migration through host cells, indicating that *AC*α is necessary to induce high levels of exocytosis and confirming the essential role of the cAMP signaling pathway in this process. Complementation of the genetically deficient parasites with the *AC*α gene confirms that the defect in exocytosis and infection observed in *PbAC*α- sporozoites is caused by deletion of the *AC*α gene and not by other modifications resulting from the genetic manipulations of these parasites.

Two genes with high homology to ACs have been identified in the *Plasmodium* genome: *AC*α and *AC*β [25]. ACα activity as an AC has been demonstrated for *P. falciparum*, where the catalytic domain was expressed independently [22]. A second putative AC gene, called *AC*β, has been identified in the *Plasmodium* database. We tried to generate *AC*β-deficient parasites; however the *AC*β gene seems to be essential for the asexual blood-stages of *Plasmodium*.

*AC*α- sporozoites are able to stimulate exocytosis in response to the permeant analogue of cAMP, but not to forskolin, the activator of ACs, confirming that the defect is caused by the lack of a functional AC and can be compensated by artificially increasing intracellular concentrations of cAMP. The results obtained with *PbAC*α- sporozoites also suggest that ACα is sensitive to forskolin stimulation, as the increase in exocytosis induced by this drug is lost in the genetically deficient sporozoites. Since AC activity is insensitive to forskolin in asexual blood-stages [35] and ACβ is preferentially expressed in this stage of the parasite cycle [25], it seems likely that ACβ, rather than ACα, is required for cAMP formation during erythrocyte infection. We also found that the growth of *PbAC*α- parasites in the asexual blood-stages was indistinguishable from control, consistent with the lack of activity of ACα during this stage.

Interestingly, the *AC*α gene contains a N-terminal domain with high homology to voltage-gated K^+^ channels. Other apicomplexans and also the ciliates *Paramecium* and *Tetrahymena* have an *AC*α gene homologous to the one in *Plasmodium* [23]. In *Paramecium* it has been demonstrated that the purified ACα protein also has K^+^ channel activity, and the generation of cAMP is regulated by K^+^ conductance [24]. Although functional K^+^ channel activity has not been demonstrated for ACα in *Plasmodium*, our results are consistent with a role for K^+^ conductance in sporozoite exocytosis. Uracil derivates do not induce exocytosis in K^+^ free medium, but activation of AC with forskolin or addition of the permeant analogue of cAMP overcomes the requirement for extracellular K^+^. Therefore, it seems likely that increased K^+^ permeability may induce activation of ACα and synthesis of cAMP.

## Materials and Methods

### Host cells and parasites

Hepa 1-6 (ATCC CRL-1830), a hepatoma cell line derived from a C57L/J mouse, which is efficiently infected by rodent malaria parasites [36] was used for *in vitro* hepatocyte infections. *Plasmodium yoelii yoelii* sporozoites (cell line 17× NL), *P. berghei* ANKA *wt* and *spect-1* deficient sporozoites [15] and the NF54 isolate [37] of *P. falciparum* were used to produce sporozoites in *A. stephensi* mosquitoes. Salivary glands were dissected from the mosquitoes. The *P. falciparum* sporozoites were extracted from the salivary glands, purified, and cryopreserved. Prior to being used in assays, the sporozoites were thawed and suspended in RPMI medium.

### Uracil derivatives

Exocytosis was induced by incubation of sporozoites with a mixture of the physiological concentrations of uracil derivatives (ICN Biomedicals) consisting of 180 µM uracil, 280 µM uridine, 300 µM uracil monophosphate (UMP), 50 µM uracil diphosphate (UDP) and 30 µM uracil triphosphate (UTP) was prepared in RPMI 1640 and pH adjusted to 7.

### Regulated exocytosis

Sporozoites (10^5^ *P. yoelii*, *P. berghei* or 5 × 10^4^ *P. falciparum*) were centrifuged for 5 min at 1800 × *g* on glass coverslips before addition of uracil derivatives or conditioned medium. After incubation at 37°C for 1 h, sporozoites were fixed with 1% paraformaldehyde for 10 min (non-permeabilizing conditions) before staining for surface TRAP/SSP2 with the monoclonal antibody (F3B5) for *P. yoelii*, *Pf*SSP2.1 for *P. falciparum* [38] and a specific TRAP/SSP2 rabbit anti-serum for *P. berghei*. Sporozoite regulated exocytosis was quantified as the percentage of total sporozoites that present a TRAP/SSP2 stained ‘cap’ in their apical end. Results are expressed as the average of triplicate determinations counting at least 50 sporozoites for each condition. Background level exocytosis was measured by staining sporozoites after dissection from mosquitoes, before incubation *in vitro*. Background exocytosis was always lower than 8% and was subtracted from all values. All experiments were performed twice showing similar results.

### Western blot

4 × 10^5^ *P. yoelii* sporozoites were incubated alone or with the different exocytosis stimuli for 1 h at 37°C before spinning at 20,000 *g* for 10 min. The supernatants were collected and separated in a 7.5% gel in reducing conditions. After semi-dry transfer to a PDVF membrane, proteins were stained with anti-*P. yoelii* MTIP antiserum followed by anti-rabbit conjugated to horseradish peroxidase. Bound antibodies were detected by chemiluminescence using ECL (GE Healthcare Bio-Sciences).

### Drug treatments

Sporozoites (10^5^) were incubated with 100 µM forskolin, 100 µM MDL-12.330A, 500 µM 8Br-cAMP, 10 µM H89, 30 µM genistein, 100 nM charybdotoxin, 50 µM SQ22536, 50 µM 2′, 5′-Dideoxyadenosine, 5 µM Adenosine 3′, 5′-cyclic monophosphorothioate 8Br-Rp-isomer, 1 nM margatoxin, 20 µM BAPTA, ionomycin 1 µM (all from Calbiochem) before addition or not of uracil derivatives for 1 h, followed by fixation and quantification of exocytosis. For exocytosis assays sporozoites were pretreated with the drug for 15 min and concentrations were kept constant throughout the experiment. For infection and migration, treatment with drugs was performed for 15 min before washing and spinning sporozoites on Hepa1-6 cells grown on coverslips placed in 24-well dishes containing 1 ml of culture medium/well. For assays in K^+^-free medium: 10^5^ *P. yoelii* sporozoites were incubated for 45 min in regular medium (RPMI 1640, that contains 5.3 mM KCl and 100 mM NaCl), K^+^-free medium (modified RPMI 1640 with no KCl and 110 mM NaCl to maintain osmolarity) in the presence or absence of stimulus, before fixation and quantification of exocytosis. To assay sporozoites viability after incubation in K^+^-free medium, sporozoites centrifuged at 20,800 g and resuspended in regular medium with uracil derivatives to induce exocytosis. All experiments were performed twice showing similar results.

### Intracellular cAMP levels

Intracellular levels of cAMP in *P. yoelii* sporozoites were determined using a cAMP Biotrack Enzymeimmunoassay system from Amersham Bioscience. For each sample 2 × 10^6^ *P. yoelii* sporozoites were incubated with uracil derivatives for 45 min at 37°C. The experiment was performed twice showing similar results.

### Migration through cells and infection

Sporozoites (10^5^ sporozoites/coverslip) were added to monolayers of 2 × 10^5^ Hepa1-6 cells for 1 h in the presence of 1 mg/ml of rhodamine-dextran lysine fixable, 10,000 MW. Sporozoites breach the plasma membrane of host cells during migration and as a result fluorescent dextran enters in their cytosol, allowing detection of wounded cells [17]. Cells were washed and incubated for another 24 hours before fixation and staining of infected cells with the mAb (2E6) recognizing HSP70 to detect infected cells [39], followed by anti-mouse IgG-FITC antibodies. Migration through host cells is quantified as percentage (or total number) of dextran-positive cells. Infection was quantified as the number of infected cells per coverslip or per 50 microscopic fields. For transwell filter assays Hepa1-6 cells (5×10^5^) were cultivated on 3 µm pore diameter Transwell filters (Costar, Corning, New York) until they form a continuous monolayer. Empty coverslips were placed underneath the filters. *P. berghei* sporozoites (2×10^5^) were added to filter insets containing Hepa1-6 cells. Coverslips were fixed after 2 h of incubation with sporozoites, before staining for surface TRAP/SSP2. All experiments were performed twice showing similar results.

### Determination of live/dead sporozoites with propidium iodide

*P. yoelii* sporozoites were incubated with the indicated drugs for 20 min before addition of propidium iodide (1 µg/ml) for 10 min. Sporozoites were washed and observed directly with a fluorescence microscope. Propidium iodide positive sporozoites were considered dead and quantified. At least 100 sporozoites were counted in each condition.

### Motility of live sporozoites

Live *P. yoelii* sporozoites were observed directly under the microscope in a heated stage at 37°C before or after addition of different stimuli. As control, the same volume of medium with the same solvent used for the stimuli was added. At least one hundred sporozoites were counted in each condition and they were classified as immobile, twisting or gliding, depending on their type of motility observed.

### Generation of the *PbAC*α- parasite lines

To disrupt the *AC*α locus an *AC*α replacement vector was constructed in vector b3D.D_T_.^^^H.^^^D_b_ (pL0001, MRA-770) containing the pyrimethamine-resistant *Toxoplasma gondii* (*tg*) *dhfr*/*ts* gene. To complement *AC*α into the genome of *PbAC*α- parasites, a vector was constructed with the human (*h*) *dhfr* selectable marker and two fragments of 4.3kb (5′) and 0.5 kb (3′) of the *AC*α gene of *P. berghei*. The linearized vector can integrate in *AC*α. Further details are described in Fig. 5. *P. berghei-ANKA* (clone 15cy1) was used to generate *PbAC*α-parasites. Transfection, selection, and cloning of *PbAC*α- parasites was performed as described [40]. Two clones (C1 and C2) were selected for further analysis. *PbAC*α- C1 parasites were transfected with the complement vector to create *AC*α- complement. Selection of transformed parasites was performed by treating infected animals with WR99210 (20 mg/kg bodyweight) as has been described [41]. One parasite clone (Cmp) in which the *AC*α gene was integrated into the *AC*α locus was selected for further analysis. Correct integration of constructs into the genome of transformed parasites was analyzed by RT-PCR and Southern analysis of restricted DNA. PCR on DNA of *WT* and *AC*α^−^ parasites was performed by using primers specific for the *WT* 5′ (flG1F 5′-AGCGCATTAGTTTATGATTTTTG-3′ and flG1R 5′-TTGTGAATTAGGGATCTTCATGTC-3′; amplifying a fragment of 0.7 kb) and *WT* 3′ (flG2F 5′-ATGCGCAAACCCGTTAAAT-3′ and flG2R 5′-TTTGATTCATTCCACTTTCCA-3′; amplifying fragment of 0.7 kb) and disrupted 5′ (flG1F and Pb103 5′-TAATTATATGTTATTTTATTTCCAC-3′; amplifying a fragment of 0.8 kb) and disrupted 3′ (flG2R and Pb106a 5′-TGCATGCACATGCATGTAAATAGC-3′; amplifying fragment of 0.9 kb) locus. PCR on DNA of complement was performed by using primers specific for INT3′ (Pb106a and flG4R 5′-GCAGAGAGAGCGTTAAAAACTATTG-3′, amplifying a fragment of 1.0 kb). RT-PCR was performed on RNA isolated from *WT* sporozoites. Primers 02-F (5′-AGGGTGACATTGAAGGGATG-3′) and 02-R (5′-ATTCCTCGGGATATTCCACC-3′) were used to amplify cDNA or genomic DNA derived from the *PbAC*α gene, amplifying a fragment of 270 bp and 658 bp, respectively.

### Genomic Southern hybridization

Genomic DNA of *P. berghei* (2 µg) was digested with HincII / EcoRI or NheI / EcoRI, separated on 0.9% agarose gel and then transferred onto a nylon membrane. DNA probe was labeled with digoxigenin using the DIG PCR labeling kit (Roche Diagnostics) using genomic DNA as template with the following primer pair, 5′-TCCTTCGTGGAATTTACACTTG-3′ and 5′-CCAGACGAGGAACTAATGCAG-3′. Signals were detected using the DIG/CPSD system (Roche Diagnostics).

### Phenotype analysis of the *PbAC*α- parasite during blood stage and mosquito stage development

Parasitemia in mice was determined by examination of a Giemsa-stained blood smear. Oocyst formation and sporozoite development were quantified in infected *Anopheles stephensi* mosquitoes as described [42]. The number of salivary gland sporozoites per mosquito was determined by dissecting salivary glands from 10 infected mosquitoes in each condition [43]. Blood stage infections were studied in mice (male Swiss Webster or C57/Bl6 mice, 20–25 g) infected with 200 µl of blood at 0.5% parasitemia. Experiment was performed twice showing similar results.

### Gliding motility of sporozoites

Gliding motility of sporozoites was analyzed by counting the average number of circles performed by single sporozoites [44]. Sporozoites (2 × 10^4^) were centrifuged for 10 min at 1,800 × *g* onto glass coverslips previously coated with anti-CS 3D11 antibody, followed by incubation for 2 h at 37°C and staining with biotin-labeled 3D11 antibody followed by incubation with avidin-FITC for sporozoite and trail visualization. Quantification was performed by counting the number of circles performed by 100 sporozoites in three independent coverslips. When indicated 3% mouse albumin was present in the assay.

### Transwell filter assays

Hepa1-6 cells were cultivated on 3 µm pore diameter Transwell filters (Costar, Corning, New York) until they form a continuous monolayer. Empty coverslips were placed underneath the filters. Sporozoites (2×10^5^) were added to filter insets containing Hepa1-6 cells or no cells. Coverslips were fixed after 2 h of incubation with sporozoites, before staining for surface TRAP to determine exocytosis. Experiment was performed twice showing similar results.

### Sporozoite infectivity *in vivo*

Groups of three C57/Bl6 mice were given i.v. injections of 20,000 sporozoites. 40 h later, livers were harvested, total RNA was isolated, and malaria infection was quantified using reverse transcription followed by real-time PCR [31] using primers that recognize *P. berghei*–specific sequences within the 18S rRNA 5′-AAGCATTAAATAAAGCGAATACATCCTTAC and 5′-GGAGATTGGTTTTGACGTTTATGT. Experiment was performed three times showing similar results.

### Accession numbers/ID numbers for genes and proteins

*P. falciparum AC*α: UniProtKB/TrEMBL accession number: Q8I7A1. PlasmoDB identifier: PF14_0043

*P. berghei AC*α: PlasmoDB identifier: PB001333.02.0. Complete *PbAC*α sequences (contig 1047, 5680) were retrieved from Sanger sequencing genomics project. *P. falciparum* PKA: PlasmoDB identifier PFI1685w.

## Supporting Information

Figure S1 Exocytosis of TRAP occurs in the apical end of sporozoites. *P. berghei* sporozoites were incubated on coverslips coated with anti-CS antibodies for 20 min before addition of forskolin. After another 30 min, sporozoites were fixed and stained for CS protein.
(5.64 MB TIF)

Figure S2 Control for sporozoite lysis. *P. yoelii* sporozoites (4 × 10^5^) were incubated for 1 h with UD, forskolin (FSK) or 8Br-cAMP. Culture media (upper panel) and pellet containing sporozoites (lower panel) were analyzed by Western blot against myosin A tail domain interacting protein (MTIP), which is localized to the inner membrane complex. A unique band at 25 kDa was found.
(1.20 MB TIF)

Figure S3 Motility of sporozoites before and after exocytosis. Live *P. yoelii* sporozoites were observed directly under the microscope before or after addition of forskolin (A) or UD (B). Sporozoite motility was classified as immobile, twisting or gliding. There is a clear shift in sporozoite motility profile from gliding to immobile at later times after addition of the stimuli. As expected, a certain decrease in motility is observed over time even in control sporozoites, however, the decrease induced by the exocytosis stimuli is significantly more pronounced. No significant changes were observed in twisting motility.
(1.23 MB TIF)

Table S1 Determination of sporozoite viability after drug treatments. *P. yoelii* sporozoites were incubated in the different conditions indicated. Dead sporozoites were quantified using propidium iodide staining. An untreated control was performed for each condition because the background level of dead sporozoites may vary on each batch of dissected mosquitoes.
(1.05 MB TIF)
