# Supplementary figures and images for: Adenylyl Cyclase α and cAMP Signaling Mediate Plasmodium Sporozoite Apical Regulated Exocytosis and Hepatocyte Infection
Source: PLoS Pathog. 2008 Feb 29;4(2):e1000008. doi: 10.1371/journal.ppat.1000008 (PMC2279260; doi:10.1371/journal.ppat.1000008)

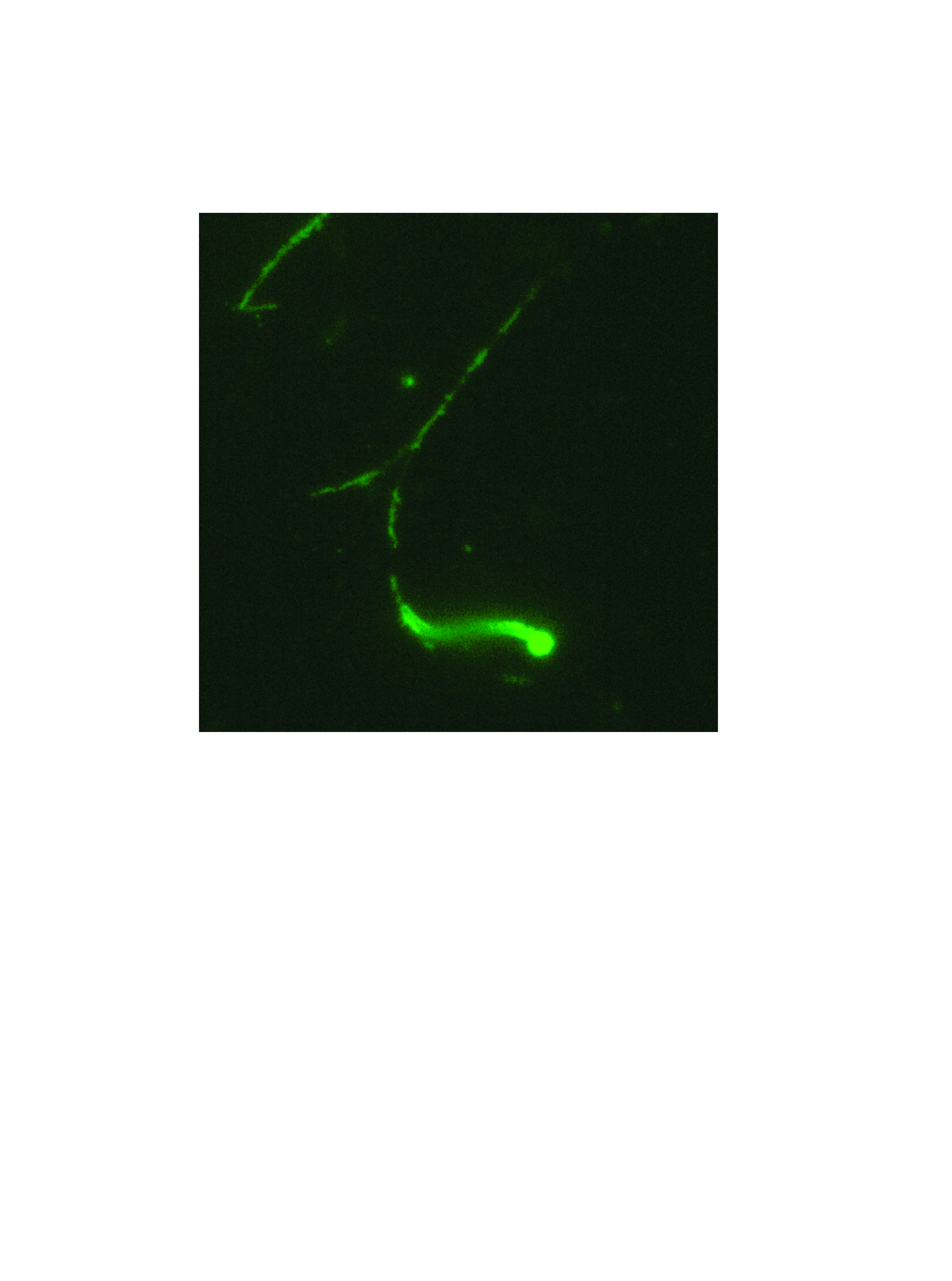

Supplement: Figure S1 — Exocytosis of TRAP occurs in the apical end of sporozoites. P. berghei sporozoites were incubated on coverslips coated with anti-CS antibodies for 20 min before addition of forskolin. After another 30 min, sporozoites were fixed and stained for CS protein. (5.64 MB TIF) [file ppat.1000008.s001.tif]

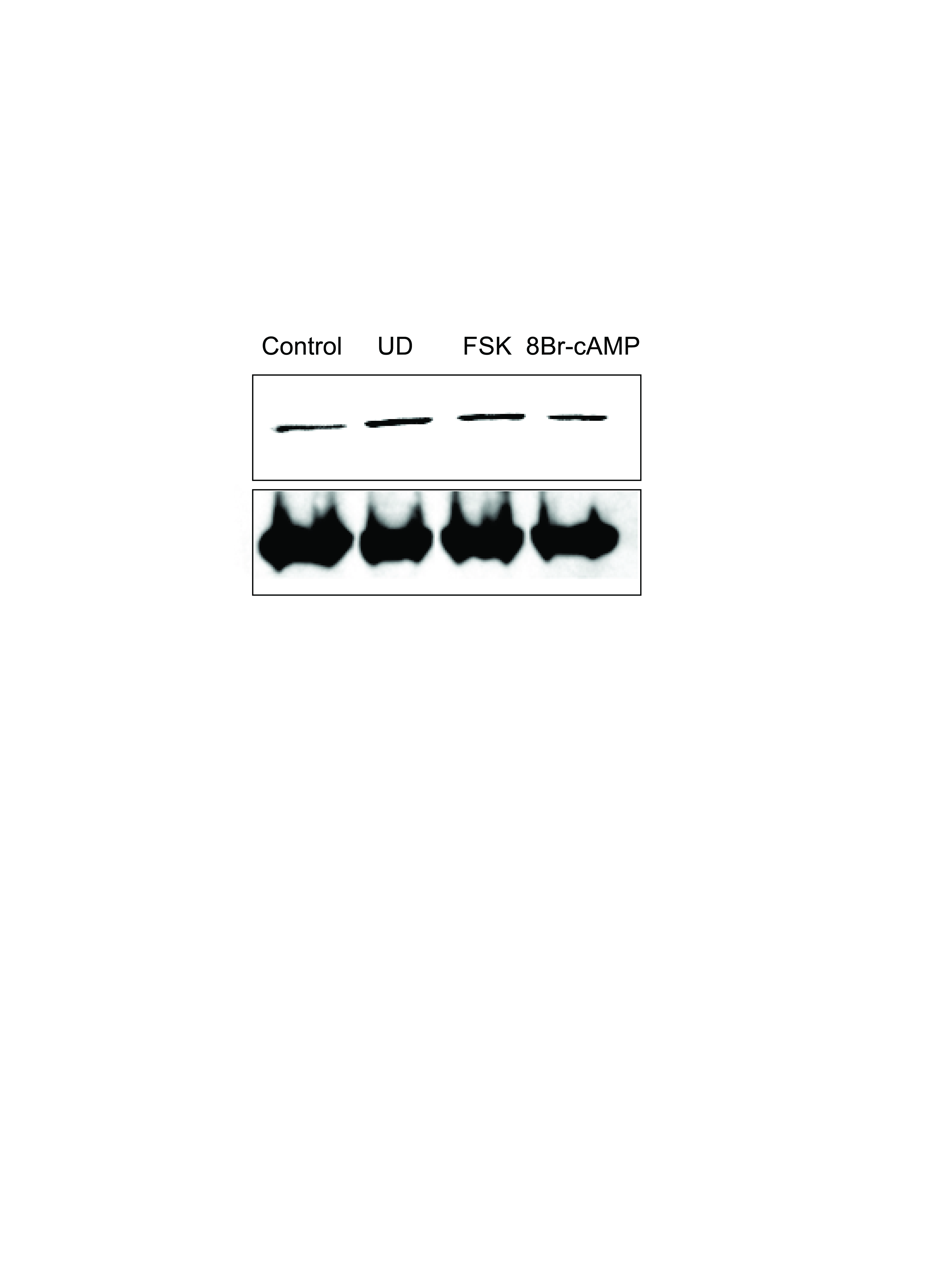

Supplement: Figure S2 — Control for sporozoite lysis. P. yoelii sporozoites (4 × 10^5^) were incubated for 1 h with UD, forskolin (FSK) or 8Br-cAMP. Culture media (upper panel) and pellet containing sporozoites (lower panel) were analyzed by Western blot against myosin A tail domain interacting protein (MTIP), which is localized to the inner membrane complex. A unique band at 25 kDa was found. (1.20 MB TIF) [file ppat.1000008.s002.tif]

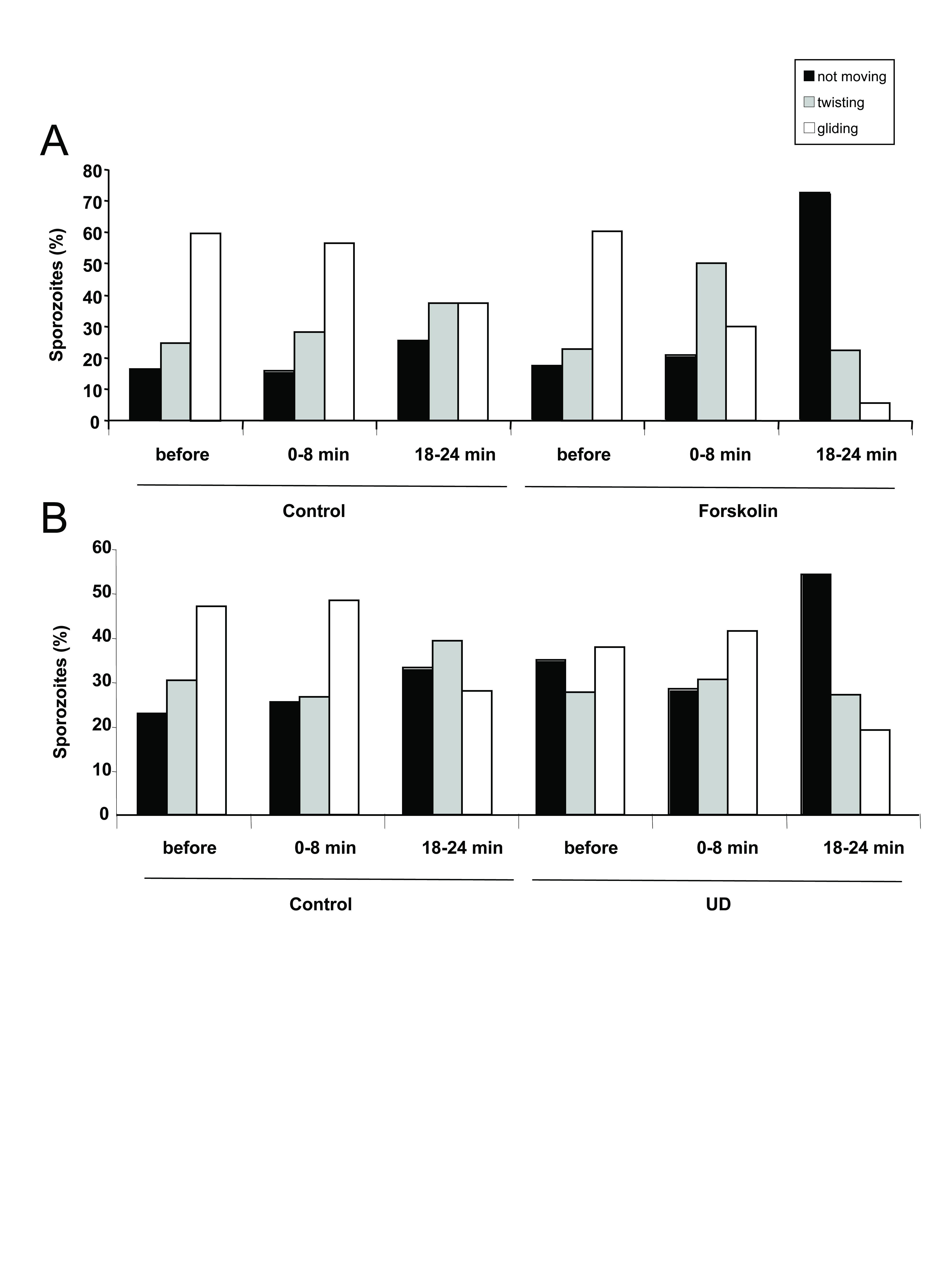

Supplement: Figure S3 — Motility of sporozoites before and after exocytosis. Live P. yoelii sporozoites were observed directly under the microscope before or after addition of forskolin (A) or UD (B). Sporozoite motility was classified as immobile, twisting or gliding. There is a clear shift in sporozoite motility profile from gliding to immobile at later times after addition of the stimuli. As expected, a certain decrease in motility is observed over time even in control sporozoites, however, the decrease induced by the exocytosis stimuli is significantly more pronounced. No significant changes were observed in twisting motility. (1.23 MB TIF) [file ppat.1000008.s003.tif]

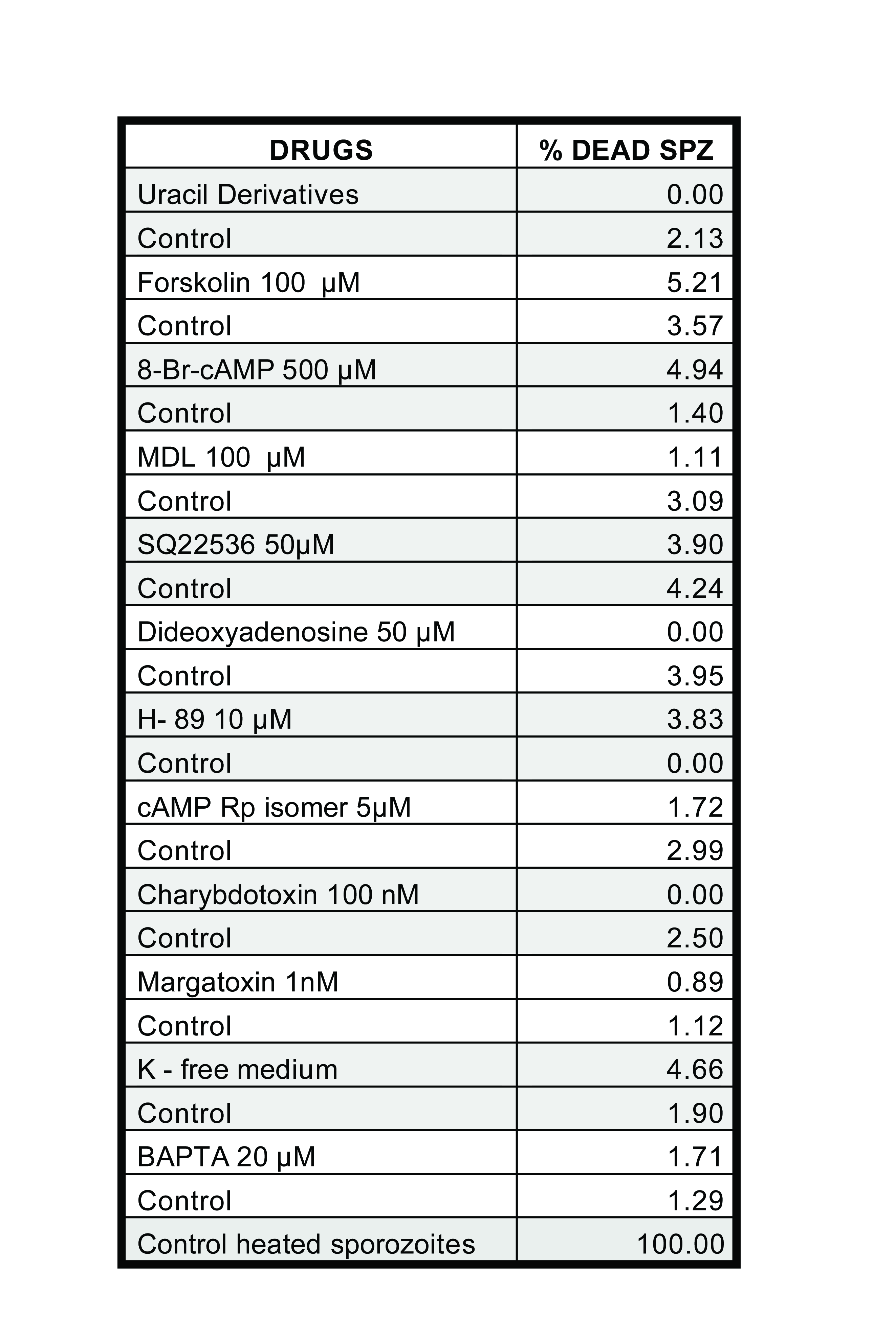

Supplement: Table S1 — Determination of sporozoite viability after drug treatments. P. yoelii sporozoites were incubated in the different conditions indicated. Dead sporozoites were quantified using propidium iodide staining. An untreated control was performed for each condition because the background level of dead sporozoites may vary on each batch of dissected mosquitoes. (1.05 MB TIF) [file ppat.1000008.s004.tif]
